# Zolfino landrace (*Phaseolus vulgaris* L.) from Pratomagno: general and specific features of a functional food

**DOI:** 10.3402/fnr.v60.31792

**Published:** 2016-07-12

**Authors:** Francesco Balestri, Rossella Rotondo, Roberta Moschini, Mario Pellegrino, Mario Cappiello, Vito Barracco, Livia Misuri, Carlo Sorce, Andrea Andreucci, Antonella Del-Corso, Umberto Mura

**Affiliations:** 1 Department of Biology, University of Pisa, Pisa, Italy; 2 Interdepartmental Research Center Nutrafood ‘Nutraceuticals and Food for Health’, University of Pisa, Pisa, Italy; 3 Department of Translational Research on New Technologies in Medicine and Surgery, University of Pisa, Pisa, Italy

**Keywords:** Zolfino bean, Phaseolus vulgaris, aldose reductase, sorbitol dehydrogenase, polyol pathway

## Abstract

**Background:**

The Zolfino bean is a variety of *Phaseolus vulgaris*, which is cultivated in a limited area of Tuscany, Italy, and is widely appreciated for its flavor and culinary uses.

**Objectives:**

A yellow Zolfino landrace cultivated in the Leccio-Reggello area was characterized and compared with three other varieties of *Phaseolus vulgaris* (i.e. the Borlotto, Cannellino, and Corona beans) in terms of its general features and potential as an antioxidant/anti-inflammatory agent.

**Design:**

The length, width, thickness, equatorial section surface, weight, volume, and seed coat section were measured in all the beans. The seed surface area was also estimated by an original empirical method. The ability of the different beans to interfere with the enzymes of the polyol pathway (that is, aldose reductase (AR) and sorbitol dehydrogenase) was tested using the supernatant after soaking the beans at room temperature and after thermal treatment, which simulated the bean-cooking process in a controlled fashion.

**Results:**

Concerning the general features, Zolfino was comparable with other beans, except Corona, in terms of surface–volume ratio, which possesses the lowest tegument thickness. Moreover, Zolfino appears the most effective in inhibiting AR activity. The inhibitory ability is unaffected by thermal treatment and appears to be associated with compound(s) present in the coat of the bean.

**Conclusions:**

The ability of Zolfino to inhibit AR, thus reducing the flux of glucose through the polyol pathway, highlights the features of Zolfino as a functional food, potentially useful in treating the dysfunctions linked to the hyperactivity of AR, such as diabetic complications or inflammatory responses.

The Zolfino bean is a variety of *Phaseolus vulgaris*, which is cultivated in the area between Arezzo and Florence in Tuscany, Italy. Zolfino has a thin coat, which means that it does not need to be pre-treated before cooking. In addition, its soft texture, special flavor, sweet taste, as well as digestibility make this bean an excellent food, appreciated for long, and a perfect complement to the traditional Tuscan cuisine (1). The Zolfino is a bush-type bean that is particularly sensitive to temperature, humidity, and soil conditions. The passion and business acumen of a few farmers have saved this variety, as well as other bean landraces, from extinction (2).

The basic nutrition parameters for the Zolfino bean, such as its protein or trypsin inhibitor content, are not complete or adequate characterizations (2). More specifically, although not exclusively, the Zolfino's pattern of secondary metabolites and antioxidant properties (3) may mean that it can be officially categorized as a functional food. The flavonoid content and the characterization of a number of Zolfino phenotypes, defined by the colors of the coat (namely, yellow, tobacco, and black), have been reported (4). Although differently represented in the three landraces, glycosylated kaempferol derivatives have been identified as the major components in alcoholic extracts, while the isoflavones daidzein and genistein have also been reported to a much lesser extent.

Essentially, the same pattern of flavonoid derivatives has been identified in 10 different analyzed ecotypes of *Zolfino del Pratomagno* grown in different areas of Arezzo (5). The variability in the composition of flavonoids observed among these samples, which was also observed among samples from the same area harvested over a 3-year period, highlights how these molecules respond dynamically to environmental and meteorological changes.

In order to extend the ability of the Zolfino bean to interact with biotargets linked to human oxidative stress conditions, the susceptibility to inhibition by components of the bean of the two polyol pathway enzymes, namely aldose reductase (AR) and sorbitol dehydrogenase (SDH), was evaluated. AR, which is the first enzyme of the pathway, catalyzes the NADPH-dependent reduction of glucose and a number of different aldehyde compounds (6). SDH, the second and last enzyme of the polyol pathway, removes the sorbitol generated by AR through an NAD^+^ dependent oxidation leading to fructose (7). As it is involved in the etiology of diabetic complications and in the NFkB-linked inflammatory signaling (8), AR is also involved in the detoxification from cytotoxic aldehydes generated from membrane peroxidative processes (9, 10). AR has thus received much attention as a target of inhibitors, capable of counteracting and/or ameliorating the pathological states linked to hyperglycemic conditions (11–13).

We characterized and compared a yellow Zolfino landrace cultivated in the Leccio-Reggello area (Tuscany) with three other varieties of *Phaseolus vulgaris* (i.e. the Borlotto, Cannellino, and Corona beans) in terms of its general features and its inhibitory potential on the polyol pathway enzymes.

## Materials and methods

### Materials

D,L-Dithiothreitol (DTT), EDTA, *β*-D(−) fructose, bovine serum albumin (BSA), NADH, and sheep liver SDH (E.C. 1.1.1.14, 40 U/mg) were obtained from Sigma-Aldrich (Saint Louis, MO, USA). NADPH and L-idose were supplied by Carbosynth (Compton, England); YM10 ultrafiltration membranes were obtained from Merck-Millipore (Darmstadt, Germany). The electrophoretic equipment was from Bio-Rad Laboratories (Hercules, CA, USA). All other chemicals were reagent grade. Molecular-weight markers for SDS-PAGE were obtained from Thermo Fisher Scientific (Waltham, MA, USA)**.

Dry beans of the yellow Zolfino landrace were obtained from the farm Agostinelli Mario in Leccio-Reggello (Florence, Italy; 43° 42’ N, 11° 27’ E), and their authenticity was confirmed by comparing their features with those registered in the ‘Regione Toscana’ germplasm data bank (access VE_027): www.germoplasma.arsia.toscana.it/. Dry beans of the Borlotto (also referred to as the ‘cranberry’ bean), the Cannellino (also referred to as the ‘alubia’ bean), and the Corona (also referred to as the ‘sweet white runner’ bean) are in the National Register under horticultural varieties access number 307, 301, and 386, respectively (www.sementi.it/articoli/164/registro-nazionale-varieta-specie-ortaggi), and were obtained from the market. The Borlotto and Cannellino beans were from Frantoio Oleario Bartolini Emilio s.r.l., Arrone, Terni, Italy; the Corona beans were from Pedon SpA, Molvena, Vicenza, Italy.

### Methods

#### Bean size evaluation

The length (*L*), width (*g*), and thickness (*p*) of the beans were measured by a vernier caliper with a precision to 0.01 mm. The perimeter and area of the equatorial section of the beans were quantitated on a scanned image of 30 beans by Image Measurement software KLONK (Ringsted, Denmark). The area was also measured by weighing the paper of the bean image carefully cut out after scanner printing. The actual surface was obtained by a surface standard reference scanned with the beans.

Bean weight was determined by weighing 20 independent samples, each composed of 10 randomly chosen beans. The weight of each sample was divided by 10 and the values averaged.

Bean volume was determined by evaluating the water displacement by the addition of 20 independent samples, each composed of 10 randomly chosen beans. The volume of each sample was divided by 10 and the values averaged.

#### Bean surface estimation

The bean surface was estimated by averaging the values from two different empirical approaches.

A. The beans were first assumed to be represented by a regular geometric solid (i.e. a ‘capsule’), composed of a cylinder ending with two hemispheres with the same radius of the cylinder base. The length of the capsule is the measured length of the bean, while its radius comes from the mean between the width and thickness of the bean, divided by two. Thus, the height of the cylinder that makes up the capsule is obtained by subtracting the diameter of the sphere from the measured bean length. The shape and size of this solid is then corrected by subtracting, from the original capsule volume, the volume of a slice cut in the equatorial region of the capsule. The thickness of the slice, approximated to a cylindroid, is calculated as described below.

The difference between the volume of the capsule (*V*) and the measured volume of the bean (*v*), was set as:

$$V-\nu =\Delta V=Ah\frac{g}{p}$$

in which *A* is the equatorial area of the capsule and $\frac{g}{p}$ is a factor introduced to take into account the effect of the shape of the bean on the evaluation. The virtual height of the cylindroid, that is, the thickness of the slice, *h*, is then used to evaluate the lateral area of the cylindroid. This value is then subtracted from the surface of the capsule in order to estimate the surface of the bean (see Supplementary Fig. 1, Panel A).

B. The bean is first assumed to be a cylindroid, whose base is shaped as the equatorial section of a capsule as defined above (see method A) in which the length and the width are the length and width of the bean. The height of the cylindroid is the thickness of the bean. The shape and size of this regular solid, which can be assumed to contain the bean, is then corrected as described below.

The difference between the volume of the cylindroid (V) and the measured volume of the bean (*v*) was set as:

$$\Delta V=V-\nu =p\frac{1}{2}pk\frac{p}{g}$$

in which *P* is the perimeter of the cylindroid base. Through this approach, the original cylindroid may be transformed into a new regular solid with the same width and approximately the same length as the original solid but consisting of a smaller cylindroid surrounded by a prism with a triangular base. The base and height of this triangle are *p* and *k*, respectively. Also, in this case, the factor $\frac{p}{g}$ was introduced to take into account the effect of the bean shape on the evaluation. The base of the new cylindroid will have a width of g^*^=*g*−2*k*, a radius of terminal semicircles r^*^=*g*/2 − *k*, and a length of the rectangular component l^*^=*L*−2*r*. This new solid, whose perimeter and surface of the base are P^*^=2πr^*^+2L^*^ and A^*^=π(r^*^)^2^+2r^*^L^*^, respectively, is complemented by a triangular base prism, whose exposed lateral surface S^*^ is $S^{*}=2P^{*}{\left[k^{2}+{\left(\frac{p}{2}\right)}^{2}\right]}^{\frac{1}{2}}$.

The surface of the emerging solid: *S*=2A^*^+S^*^ is then taken as an estimate of the bean surface (see Supplementary Fig. 1, Panel B).

The surface evaluation of the bean is finally obtained from the average of the surfaces measured with both methods A and B. Because of comparable errors in direct measurements (Table 1) on different beans, no error propagation analysis of measured parameters in the calculated parameters was performed.

**Table 1:** Size parameters of different beans

	Measured parameters		Zolfino	Borlotto	Cannellino	Corona
1	Weight (g)^a^		0.34±0.01	0.58±0.02	0.72±0.02	2.40±0.12
2	Weight of tegument (g)^b^		0.021±0.002	0.044±0.008	0.050±0.006	0.382±0.03
3	Tegument w/total w (w/w %)		6.18	7.59	6.94	15.91
4	Volume (cm^3^)^c^		0.27±0.02	0.47±0.04	0.55±0.02	2.58±0.04
5	Length (cm)		1.04±0.13	1.39±0.08	1.63±0.08	2.77±0.13
6	Minor section (thickness) (cm)		0.64±0.05	0.67±0.05	0.73±0.04	0.98±0.10
7	Major section (width) (cm)		0.71±0.08	0.89±0.06	0.82±0.04	1.70±0.09
8	Equatorial surface by weight (cm^2^)		0.65±0.13	0.98±0.13	1.12±0,14	3.48±0.40
9	Equatorial surface by Klonk^d^ (cm^2^)		0.55±0.08	0.91±0.10	0.97±0.11	3.44±0.45
10	Perimeter by Klonk^d^ (cm)		3.35±0.26	4.39±0.30	4.70±0.22	8.60±0.63
11	Tegument thickness	By caliper^e^	79±34	160±49	112±59	270±98
12		By image analysis	93±8	147±14	133±10	312±21
13		Density (g/cm^3^)	1.26±0.1	1.23±0.11	1.31± 0.06	0.93±0.05
	Calculated parameters					
14	Surface (cm^2^)	Method A^d^	2.1	3.2	3.7	10.8
15		Method B^d^	2.0	3.3	3.5	11.7
16		A, B average	2.0	3.2	3.6	11.3
17	Surface/volume (cm^−1^)		7.7	6.9	6.5	4.4
18	Tegument weight/surface (g/cm^2^)		0.010	0.013	0.014	0.034
19	Thickness (µm)	Referred to Zolfino	93	121	130	316
20		Referred to Corona	92	120	128	312

a 20 independent measurements of groups of 30 beans.

b 3 independent measurements of groups of 10 beans.

c 10 independent measurements of groups of 10 beans.

d See Methods.

e At least 15 independent measurements.

In order to validate the surface estimate of the beans, the Corona beans were used as a reference, because they are relatively large and their coats are easy to remove. Thus, 25 beans were cut in order to separate the cotyledons, very rapidly wetted (15–20 sec), and the teguments gently removed. In these conditions, the tegument, which comes out almost as an integer, was transversely cut in order to make it flat, kept for few minutes, gently squeezed between sheets of filter paper, and then scanned. The area was then measured as above.

#### Seed coat section measurements

In order to evaluate the contribution of the seed coat (i.e. the outer integument plus the inner integument and residual endosperm) to the total weight of the bean, the coat was gently removed. The part of the endosperm that had remained attached to the cotyledons was gently scraped and added to the removed coat. The weight of the material recovered after this approach (i.e. the tegument plus cotyledons) was never less than 99% of the weight of the integer bean.

In order to measure the thickness of the coat, parts of the tegument were gently removed to prevent any endosperm detachment and checked by optical microscopy for the absence of particles, possibly from the cotyledons, taken during the coat removal. The thickness was measured by a Vernier caliper with a 0.01-mm precision. Because of the difficulties in controlling the pressure applied on the caliper in each measurement, thickness determination was also performed by optical microscopy imaging. Tegument transverse sections were mounted on the stage of a Nikon Eclipse E600FN microscope with a 10× objective. The epi-illumination of the sample was performed with an optical fiber. Images were acquired by a CCD camera (WV-BP514E, Panasonic, Kadoma, Japan) and collected using Axon Imaging Workbench 2.2 software (Molecular Device, Sunnyvale, CA, USA), by averaging 16 frames (time of exposure 528 ms). Acquired images were processed using the open source Image-J software.

#### Assay, expression, and purification of AR

The activity of AR (E.C. 1.1.1.21) was determined at 37°C, following the decrease in absorbance at 340 nm due to NADPH oxidation (ɛ_340_=6.22 mM^−1^·cm^−1^). Briefly, the standard assay mixture (700 µL) contained 0.25-M sodium phosphate buffer pH 6.8, 0.18-mM NADPH, 0.4-M ammonium sulfate, 0.5-mM EDTA, and 4.7- mM glyceraldehyde. The oxidation rate of NADPH oxidation in the absence of the substrate was subtracted as a blank. One unit of enzyme activity is the amount that catalyzes the conversion of 1 µmol of substrate/min in the above assay conditions. These assay conditions were also adopted to evaluate the effectiveness as inhibitors of the bean extracts when 0.6-mM L-idose was used as a substrate.

The human placental aldose reductase (*h*AR) was expressed and purified as previously described (14, 15). The specific activity of purified *h*AR was 5.3 U/mg of protein. The purified *h*AR was stored at −80°C in a 10-mM sodium phosphate, buffer pH 7.0 containing 2-mM DTT with 30% (w/v) glycerol. Before use, the enzyme was extensively dialyzed against sodium phosphate buffer pH 7.0.

#### Assay of SDH

The SDH assay was performed at 37°C, essentially as previously described (16), by following the decrease in absorbance at 340 nm in a reaction mixture (700 µL) containing 0.18-mM NADH and 0.4-M D-fructose in 100-mM Tris/HCl, buffer pH 7.4. The rate of NADH oxidation measured in a parallel assay, in which the substrate was omitted, was subtracted as a blank. One unit of enzyme activity is the amount of SDH that catalyzes the oxidation of 1 µmol substrate/min in the above assay conditions.

#### Other methods

Protein concentration was determined according to Bradford (17), using BSA as a standard protein. The protein pattern of bean extracts was assessed through SDS-PAGE (18); gels were stained with 0.1% Coomassie blue R250, in 10% acetic acid and 20% ethanol. Acquired images of stained gels were processed using the open source Image-J software.

Absorption spectra were acquired through a Biochrom Libra S35PC single beam spectrophotometer (1-nm bandwidth), calibrated with water as a reference. If not otherwise specified, the absorbance data acquired within the instrumental linear dynamic range were then converted into true values by multiplying the absorbance values by the dilution factor.

All the samples of seed extracts and seed-flour extracts analyzed for both absorption spectra and enzyme inhibitory activity were centrifuged at 4°C for 1 h at 9.6×10^4^×*g* in a Beckman Optima L-90K ultracentrifuge before analysis.

Statistical analysis was performed using standard statistical software (GraphPad Instat version 3.05, San Diego, CA); the results are reported as the mean of the values±standard deviation and the number of replicates is indicated each time.

## Results and discussion

**Fig. 1:**
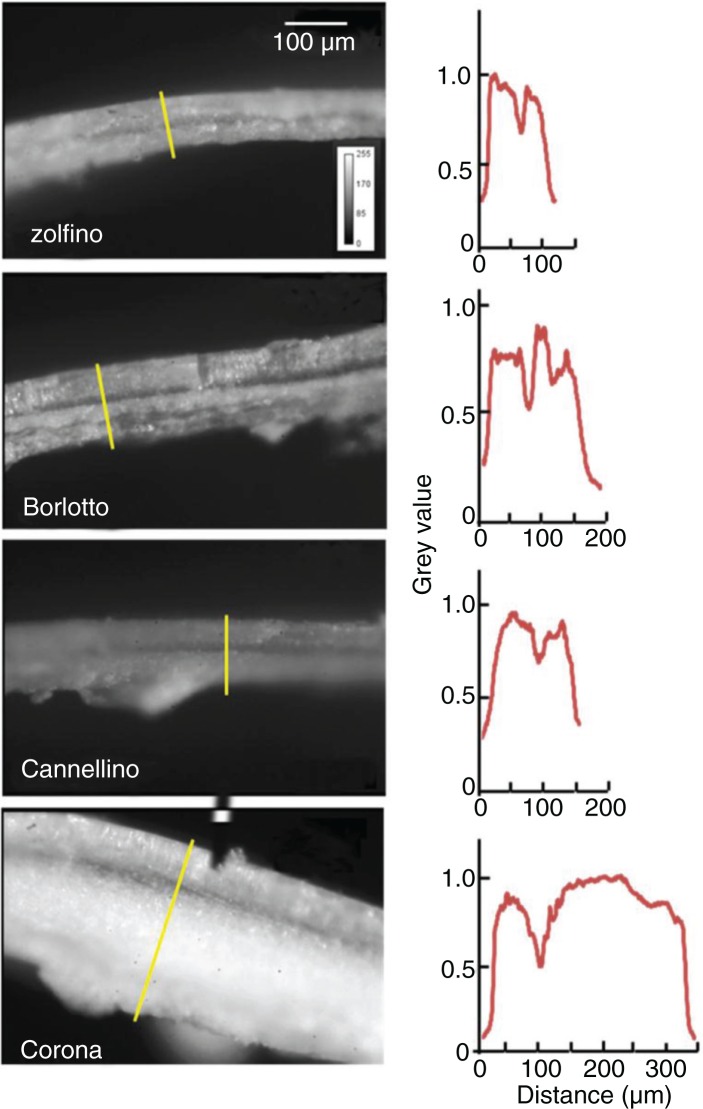
Epi-illumination micrographs of tegument transverse sections from four varieties of beans. On the right of each image, a two-dimensional graph of the pixel value as a function of the distance along the yellow line is displayed.

The distinctive size parameters of the Zolfino landrace used in this study, such as length, width, thickness, equatorial section surface, weight, and volume, were measured. The results are summarized in Table 1. The bean surface was also estimated (Table 1) as described in the ‘Methods’ section. The method was validated using the Corona bean, whose calculated surface was 11.3 cm^2^, which compares well with the measured value of 11.1±1.7 cm^2^. With this approach, the Zolfino surface was 2.0 cm^2^, which is indicative of the highest exposed surface per unit weight (5.9 cm^2^/g) and per unit volume (7.4 cm^−1^) of the tested beans. The ratios of both surface/weight and surface/volume of all the beans were similar, except for the Corona beans, whose surface/volume ratio was significantly lower (approximately 40% of that of the Zolfino bean). To quantify the thinness of the Zolfino coat, portions of the coat were used by measuring the section thickness with a caliper. The variability of the measurements (Table 1, line 11), even in the same sample, was overcome by evaluating the coat section with microscopy imaging. The typical organization of the teguments was analyzed on a digital image, through the distribution of the pixel-grade level along lines crossing the coat sections. The images of the different beans and the relative profile graphs are shown in Fig. 1. As can be seen, the endosperm is mainly responsible for the observed differences, as the inner and the outer integuments are very similar among the beans. The results of the analysis are reported in Table 1, line 12.

Using these measured values, we evaluated the tegument thickness from the weight/surface ratio of the tegument. The calculated thickness was in a good agreement with the measured values, suggesting that the beans were similar in terms of coat density. This is shown in Table 1, in which the measured coat thickness of both the Zolfino and the Corona were used as a reference value (compare lines 19 and 20 with lines 11 and 12).

**Fig. 2:**
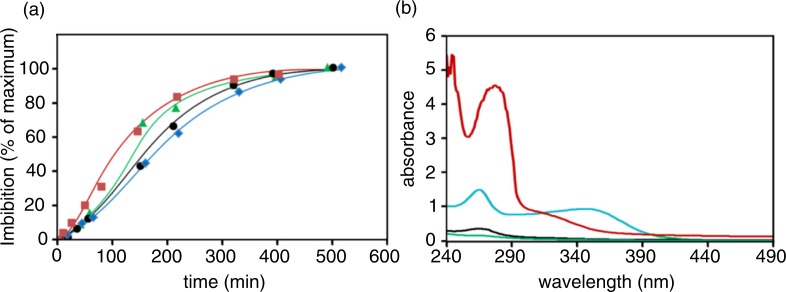
Water entry and extrusion of molecular species. Beans were suspended (0.4 g/mL) in deionized water and left at room temperature (22–25°C). Panel a: at the indicated times, the incubation medium was carefully removed and the water entry, reported as a percentage of maximal imbibition, was evaluated by the increase in bean weight. Symbols (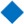), (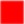), (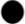), and (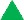) refer to Zolfino, Borlotto, Cannellino, and Corona, respectively. Panel b: the absorption spectra are reported in the UV-visible region of the imbibition medium after 1 h of incubation. The spectra were acquired directly on the incubation media except for Borlotto for which a twofold dilution in water of the medium was required; data are normalized for the dilution. Blue, red, black, and green lines refer to Zolfino, Borlotto, Cannellino, and Corona, respectively.

Despite a lower coat thickness and larger surface contact, conceivably leading to more efficient fluid entry, no correlation between the relative surface exposure and the time course of water absorption could be found. Indeed, the Zolfino bean, closely followed by the Cannellino bean, is more resistant to water entry than the Corona and Borlotto beans (Fig. 2a). This is not surprising, as water and solutes trafficking in and out of the tegument are perhaps governed by the overall structural differences in permeability and composition linked to different bean varieties (19–21).

**Fig. 3:**
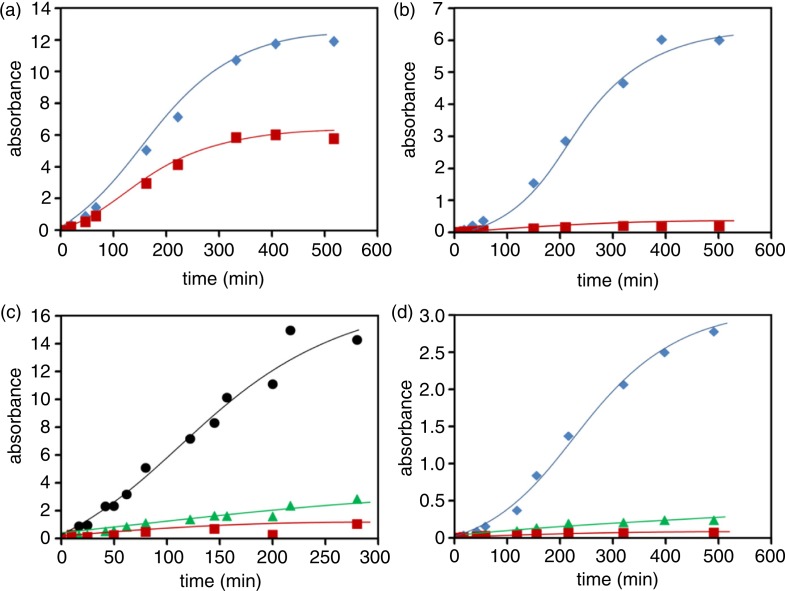
Time course of chromophores extrusion during beans imbibition. The release of bean components and incubation times are reported for different beans as the absorbance measured at distinctive wavelengths, namely 265 (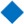), 273 (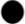), 310 (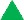), and 345 (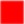) nm. Panels a, b, c, d refer to Zolfino, Cannellino, Borlotto, and Corona, respectively.

The extrusion of compounds from the beans undergoing water imbibition was monitored by absorption spectroscopy. The difference between different bean varieties is clearly shown in Fig. 2b, which reports the absorption spectra of the incubation medium of different beans after 1 h of incubation at room temperature (22–25°C). The absorption spectra obtained at different times of incubation are shown in the Supplementary Fig. 2. The difference in the extruded compounds during incubation is highlighted in Fig. 3, which reports the absorbance at different wavelengths for the different beans at different times.

**Fig. 4:**
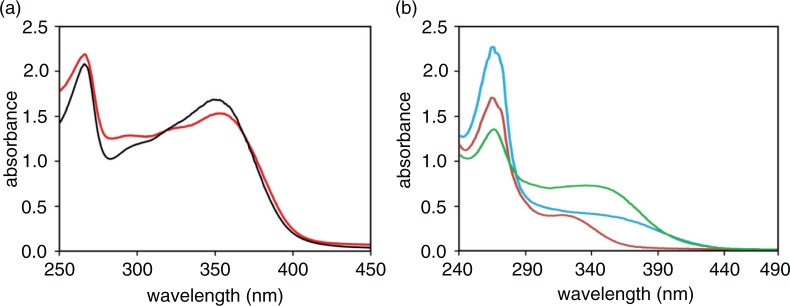
Yellow chromophores from Zolfino. Panel a: Absorption spectra of methanol extract of Zolfino tegument (the tegument from 1.5 g of seeds soaked in 3 mL methanol for 1 h at room temperature) (red line) and of 20 µM in methanol of authentic kaempferol-3O-glucoside (black line) are reported. Panel b: absorption spectra of water extract from tegument (green line), cotyledons (red line), and whole (blue line) Zolfino bean are reported. Teguments and cotyledons derived from 1.5 g of Zolfino seeds were soaked for 1 h at room temperature in 3 and 6 mL of water, respectively, and the medium analyzed. Whole seeds (1.5 g) were soaked as above in 6 mL of water and the medium analyzed.

Beside an absorption peak in the region 260–280 nm, which is common to all the bean varieties, an absorbance peak is evident in the Zolfino spectrum at approximately 350 nm, with a tail protruding over 400 nm, which is not present in other beans. Although no attempt was performed to specifically identify the yellow-colored component(s), the spectral features are consistent with those of the flavonoid family, previously identified in the Zolfino (4, 22). This is supported by the comparison, reported in Fig. 4a, of the spectra of a methanolic extract of the Zolfino coat and of a solution of authentic kaempferol 3-O-glucoside, which is the most representative flavonol present in the Zolfino bean. Thus, the ratio of the absorbance at 265 and 345 nm, observed in the water extract media, which is too high for a typical kaempferol derivative (Figs. 3 and 4b), may derive from the presence in the incubating medium of other water-soluble chromophores extruded from the cotyledons or/and from the bean coat. As shown in Fig. 4b, the absorption spectra of aqueous extracts from the integer bean, coat, and cotyledons indicate that the yellow component(s), which may be responsible for the pale yellow color of the bean, from which the name Zolfino is derived, as expected (2), is essentially from the coat. Conversely, the material that contributes most to the absorption in the 260–280-nm region appears to mainly originate from the inside of the bean embryo.

### Protein pattern

**Fig. 5:**
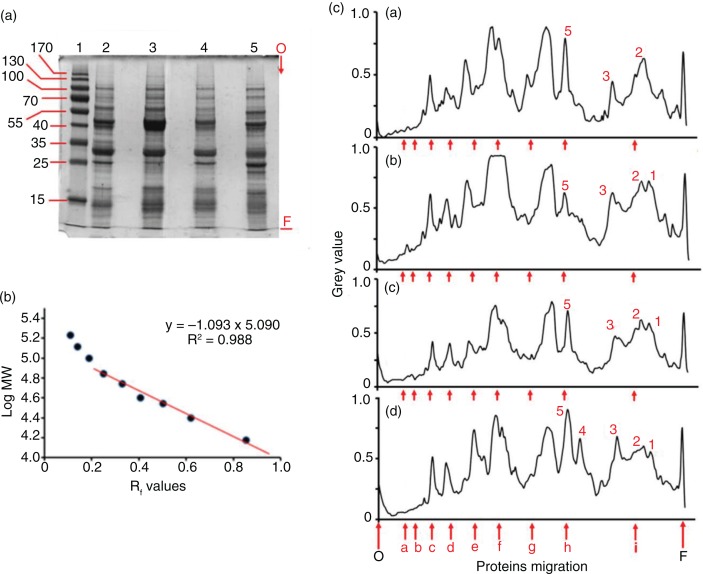
Electrophoretic pattern of water-soluble proteins from different bean varieties. Water-soluble proteins extracts from bean flours (100 mg/mL) were analyzed by SDS-PAGE on 12% acrylamide gels. 3 µg of proteins were applied to each well. Panel a: Coomassie blue–stained gel of water flour extracts of Zolfino, Cannellino, Borlotto, and Corona beans (lanes 2 to 5, respectively). Lane 1 refers to MW standard proteins. Numbers alongside the gel represent their apparent molecular weights divided by 1,000. Panel b: molecular-weight calibration curve using standard proteins (lane 1 of the stained gel). The equation reported was obtained by linear regression analysis of standards migration Panel c: aligned electropherogram profiles for Zolfino (a), Cannellino (b), Borlotto (c), and Corona (d). Arrows marked with letters *a–h* refer to the migration of MW standards reported in Panel b. Numbers on the profiles refer to specific protein bands characterizing the protein patterns. The letters O and F refer to the origin and to the front of the migration, respectively.

Various certified nutritional parameters of the Zolfino bean are reported in Table 2, together with those of other more common varieties of *Phaseolus vulgaris*. Although the reported values suggest that this bean variety is a valuable food, these parameters are limited in their ability to distinguish Zolfino from the other more common bean varieties (2). In this regard, the literature reports a wide range of values for these parameters (23, 24). Furthermore, a direct comparison of the percentage content (g/100 g dry bean) of water-soluble proteins in the bean flours (4.6±0.3, 3.8±0.3, 3.9±0.4, and 4.4±0.3, for the Zolfino, Cannellino, Borlotto, and Corona beans, respectively) does not reveal significant differences among the beans. When the comparison was extended to the electrophoretic pattern, some specific features emerged (Fig. 5). A much more detailed investigation, which is beyond the scope of this study, would be required for a complete analysis of the protein characterizations (25). However, in terms of the aligned electropherogram profiles of the beans, we found differences in the relative distributions of protein bands in the phaseolin (47,000–50,000 MW) and phytohemagglutinin (29,000–30,000 MW) regions. The triplet of bands (Fig. 5c, bands 1, 2, and 3) present in the MW range of 13,000–18,000, also appears to be a discriminating factor. In fact, these bands appear equally represented in the Cannellino and Borlotto beans, while band 1 is poorly represented or unrepresented in the Zolfino bean, and band 3 appears to prevail in the Corona bean. Other examples of particular protein patterns are the 23,400 MW band (band 4), well represented in the Corona bean, but lacking in the Zolfino, Cannellino, and Borlotto beans; and the 26,000 MW band (band 5), which is poorly represented in the Borlotto bean compared with all other beans.

**Table 2:** Some nutritional parameters of different beans

Measured parameters g/100 g dry bean	Zolfino^a^	Borlotto^b^	Cannellino^b^	Corona^b^
Proteins	23.10±1.85	19.4	23.6	23.4
Total carbohydrates	39.16±0.48	47.2	43.1	45.1
Total fat	2.43±0.10	0.8	0.8	0.9
Saturated fat	0.40±0.01	0.1	0.1	0.2
Fibers	19.50±5.85	16.5	17.2	15.2

a Values certified by ChelabSilliker Italia, a Mérieux NutriSciences company, Test Report No. 1433658.02 (2014).

b Values certified by the producer company; no standard deviation is available.

### Bio-interactions

**Fig. 6:**
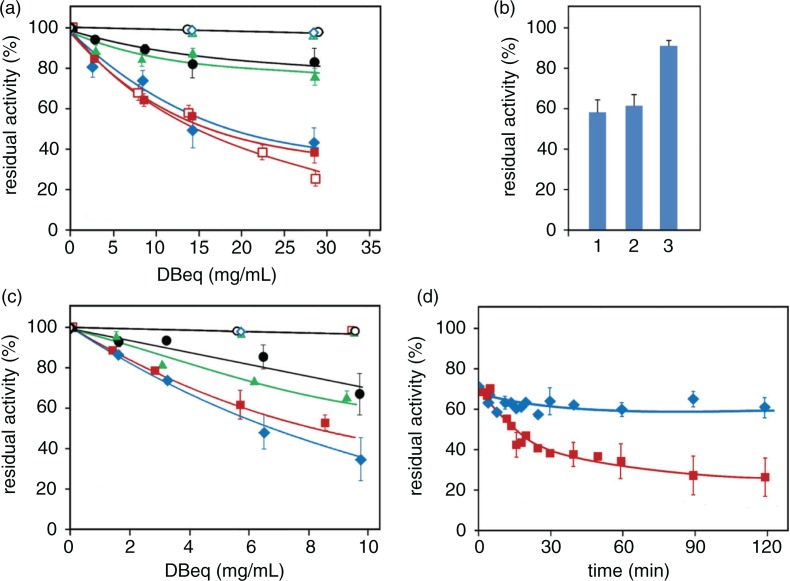
Polyol pathway enzymes inhibition by bean water extracts. The effect of different extracts from Zolfino (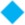, 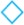), Borlotto (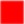, 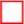), Cannellino (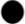, 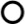), and Corona (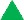, 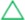) was tested on AR (closed symbols) and SDH (open symbols). The residual activity is reported as a% of the activity measured in the absence of the effector, which accounts for 8 and 3.1 mU for AR and SDH, respectively. Error bars (when not visible, these are within the symbol size) represent the standard deviation from three independent measurements. Panel a: The aqueous medium derived from an overnight incubation at room temperature of the bean seeds (400 mg/mL) was centrifuged; various amounts of the supernatants (expressed as DBeq mg/mL, see text) were added to the standard assay mixture of AR and SDH. Panel b: the whole Zolfino bean (bar 1), tegument (bar 2), and cotyledons (bar 3) were incubated overnight at room temperature in water at 200, 12, and 190 mg/mL, respectively. After centrifugation, aliquots of the supernatants (corresponding to 8.6 DBeq mg/mL) were tested for AR inhibition effectiveness. Panel c: Bean seeds were incubated (200 mg/mL) in a boiling water bath for 2 h. After the seeds had been separated, the medium was centrifuged and tested for AR and SDH inhibitory efficiency. Panel d: The aqueous medium from an overnight incubation at room temperature (400 mg/mL) was centrifuged and the supernatant incubated in a boiling water bath. At the times indicated, aliquots were withdrawn and tested (4.3 DBeq mg/mL) for AR inhibition effectiveness.

The ability of different beans to interfere with the activity of the enzymes of the polyol pathway (i.e. AR and SDH) was tested by using the supernatant of the suspending medium from the imbibition curves performed at room temperature. The inhibitory potential on *h*AR of different extracts is reported in Fig. 6a. The Zolfino and Borlotto beans were found to extrude the most active inhibitory mixtures; each displayed an IC_50_ of approximately 14.3 mg equivalents of dry bean/mL of assay mixture (DBeq mg/mL). In the same conditions, the Cannellino and Corona beans did not reach 50% inhibition, and at the maximal tested level of 28.6 DBeq mg/mL showed 17 and 23% AR inhibition, respectively. As happens with the yellow chromophores of beans (Fig. 4b), the inhibitory potential on AR activity of the extract resulting from the imbibition process also appears to be confined to the coat. This is highlighted in Fig. 6b, which reports a comparable inhibition of the water extract from the bean coat and from the whole bean. Conversely, no inhibition is displayed by the water extract of cotyledons. When SDH was used as a target enzyme, the Borlotto extract was the only one that inhibited the enzyme, with an IC_50_ of approximately 14.3 mg DBeq/mL. This feature, favoring polyalcohol accumulation, as it occurs, for example, in galactose-induced cataract (26, 27), would be deleterious for the cell. No SDH inhibition was observed with other beans at the maximal level tested of 28.6 DBeq mg/mL.

**Fig. 7:**
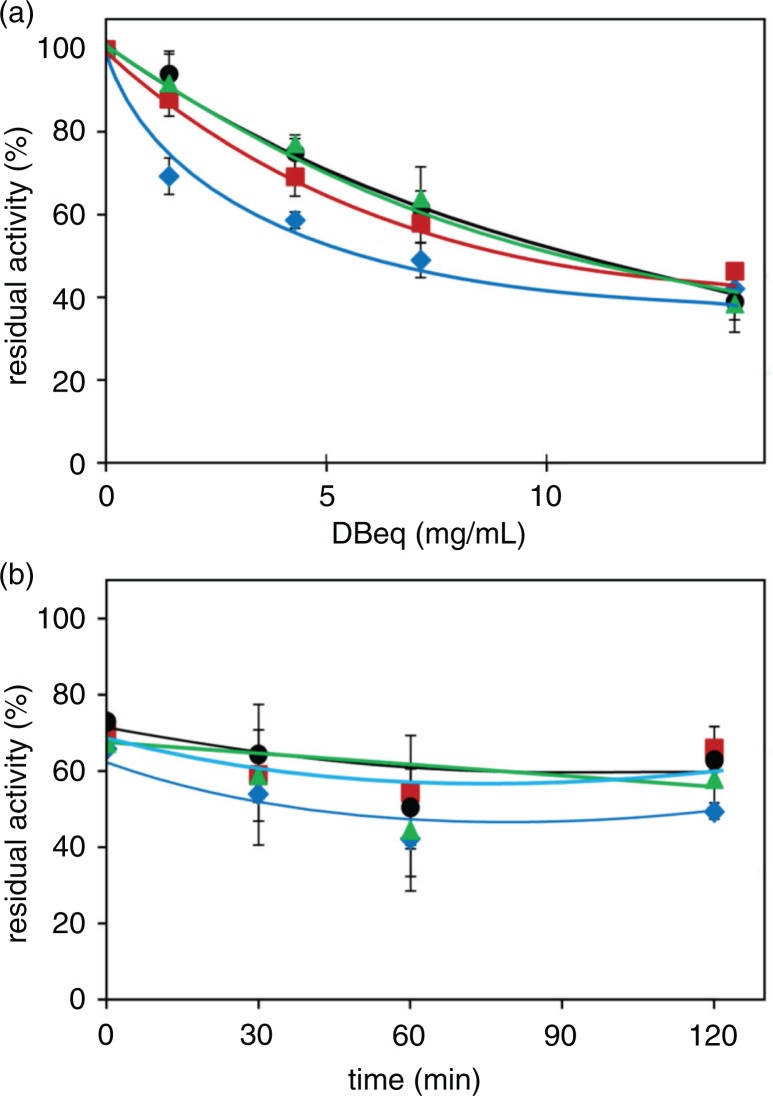
Aldose reductase inhibition by water extract of bean flours. The effect of different extracts from the flour of Zolfino (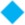), Borlotto (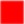), Cannellino (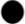), and Corona (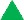) was tested on AR activity. The residual activity is reported as a percent of the activity in the absence of the effector, which accounts for 8 mU. Error bars (when not visible, these are within the symbol size) represent the standard deviation from three independent measurements. Bean seeds frozen in liquid nitrogen were powdered in a mortar, suspended (400 mg/mL) in water, and centrifuged (see Methods). Panel a: the indicated amounts of supernatants (expressed as DBeq mg/mL, see text) were used to test the AR inhibitory effectiveness. Panel b: the supernatants were subjected to incubation in a boiling water bath, and at the times indicated, aliquots were withdrawn and tested (4.3 DBeq mg/mL) for AR inhibition effectiveness.

Although, to our knowledge, no toxicity data are reported for the Zolfino bean, it is well known that beans should not be eaten raw, due to the presence of antimetabolites and toxic compounds (28). Thus, the inhibitory action of different beans was measured on the supernatant of the suspending medium of the beans kept for 2 h at 100°C in a boiling water bath (‘cooking medium’). These conditions simulate the process of beans being cooked, yet do not destroy the inhibitory potential, which actually seems to be enhanced. The cooking media of the Zolfino and Borlotto beans displayed an IC_50_ ranging from approximately 6.4 to 7.9 mg DBeq/mL. Although the inhibitory action of Cannellino and Corona beans was greater than when the incubation was performed at room temperature, it did not exceed 30–35% inhibition at the highest tested amount of approximately 10-DBeq mg/mL in the assay (Fig. 6c). The observed increase in the inhibitory ability of the beans after the thermal treatment may derive either from a more extensive release from the beans of the inhibitory species (the same or different species with respect to those extruded at room temperature) (see Supplementary Fig. 3) or from their modification under thermal treatment (see Supplementary Fig. 4). All options may also take place. In fact, the progressive increase in absorption of the cooking medium over time indicates that, as expected, cooking leads to a massive release of the internal bean components (with or without AR inhibitory action) (see Supplementary Fig. 3). On the contrary, the incubation at 100°C of the medium obtained at room temperature determined, for the Borlotto bean, both spectral changes (see Supplementary Fig. 4) and a significant increase of the inhibitory effect on AR (Fig. 6d). These effects were not observed with the Zolfino bean. Other factors must thus explain the comparable inhibitory action of the Zolfino and Borlotto beans when the whole beans were thermally treated (Fig. 6c). For example, some of the components extruded and activated, during the thermal treatment of the whole bean, may either contribute to the Zolfino inhibitory effect or mitigate the effect, as in the case of the Borlotto bean. This hypothesis, which we are currently investigating, may be supported by the results concerning the inhibitory potential of the water extracts of powdered beans (Fig. 7a). In this case, although Zolfino still appears to be the most active, all the bean extracts display a comparable AR inhibitory activity, and no marked increase of the inhibitory potential after the thermal stress was observed for any of them (Fig. 7b).

After thermal treatment, no inhibition of SDH activity was observed for any of the extracts, including the Borlotto bean (Fig. 6c). Consequently, after cooking the beans, the deleterious influence due to the inhibition of the sorbitol removal through the polyol pathway is, in any case, abolished.

**Fig. 8:**
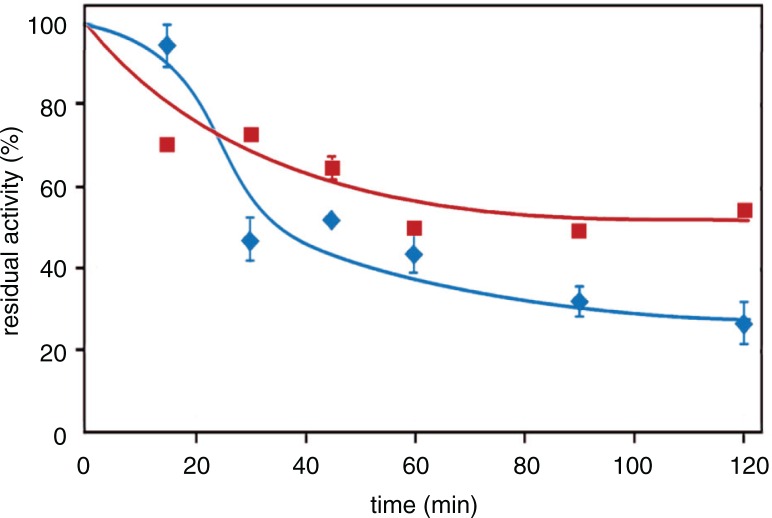
Aldose reductase inhibition by the ‘cooking media’ of Zolfino and Borlotto. The suspending media of Zolfino directly incubated at 100°C (400 mg/mL) in water, and of Borlotto incubated as above after the removal of the water in which the bean (400 mg/mL) had been soaked overnight at room temperature. Residual AR activity was measured at different cooking times using 8.6-DBeq mg/mL and is reported as a percentage of the activity in the absence of the effector, which accounts for 8 mU. Error bars (when not visible, these are within the symbol size) represent the standard deviation from three independent measurements. (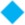) and (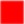) refer to Zolfino and Borlotto, respectively.

A final comparison between the Zolfino and Borlotto beans, which were the most similar in terms of AR inhibition ability, was made after following specific recommendations for cooking the beans. Thus, Fig. 8 reports the AR inhibitory potential detectable in the cooking media of Borlotto beans previously soaked in water, as recommended in many recipes, and of Zolfino beans, cooked without soaking, as is also recommended. In this case, the Borlotto beans, having lost some inhibitory potential because of the preliminary soaking, are less efficient in acting on AR.

## Conclusions

The known tenderness of the coat of the Zolfino bean, ease of cooking, excellent taste, mild flavor, and easy digestibility were good reasons for investigating this variety of bean. However, the particular pattern of the possible antioxidant metabolites shown in the Zolfino provided a further motivation for examining its properties as a functional food. In terms of the data characterizing the Zolfino with respect to other beans varieties, this study focused on the targets of AR and SDH, the two enzymes of the polyol pathway, in order to assess the potential of this bean to beneficially impact deleterious metabolic conditions. In terms of the extracts of thermally untreated beans, the AR inhibition data were useful in presenting the particular features of the different bean varieties, but the maintenance of the AR inhibitory potential of Zolfino after cooking is of special interest from a nutraceutical point of view. Zolfino's potential to reduce the flow of glucose, through the polyol pathway and without interfering with the SDH-dependent sorbitol removal, highlights that this bean can be exploited as a functional food. It suggests its ability to intervene in dysfunctions linked to the hyperactivity of AR, such as diabetic complications or/and inflammatory responses through AR-linked NF-kB cell signaling.

Further studies are required to correlate the observed AR inhibitory action of the well-defined components of raw beans, as well as those components that result from thermal treatment (that is, cooking). In this regard, work is in progress to examine the ability of different Zolfino components to differentially inhibit AR (13), which would increase its activity against diabetes complications and inflammation.

## Supplementary Material

Zolfino landrace (*Phaseolus vulgaris* L.) from Pratomagno: general and specific features of a functional food
