# Supplementary material for: Zolfino landrace (Phaseolus vulgaris L.) from Pratomagno: general and specific features of a functional food
Source: Food Nutr Res. 2016 Jul 12;60:10.3402/fnr.v60.31792. doi: 10.3402/fnr.v60.31792 (PMC4944724; doi:10.3402/fnr.v60.31792)
Supplement: Zolfino landrace (Phaseolus vulgaris L.) from Pratomagno: general and specific features of a functional food [file FNR-60-31792-s001.pdf]

Zolfino landrace (*Phaseolus vulgaris L.*) from Pratomagno: general and specific features of a functional food.

Francesco Balestri[1], Rossella Rotondo[1], Roberta Moschini[1,2], Mario Pellegrino[3], Mario Cappiello[1,2], Vito Barracco[1], Livia Misuri[1], Carlo Sorce[1,2], Andrea Andreucci,[1,2], Antonella Del-Corso*[1,2] and Umberto Mura[1,2].

Supporting Information

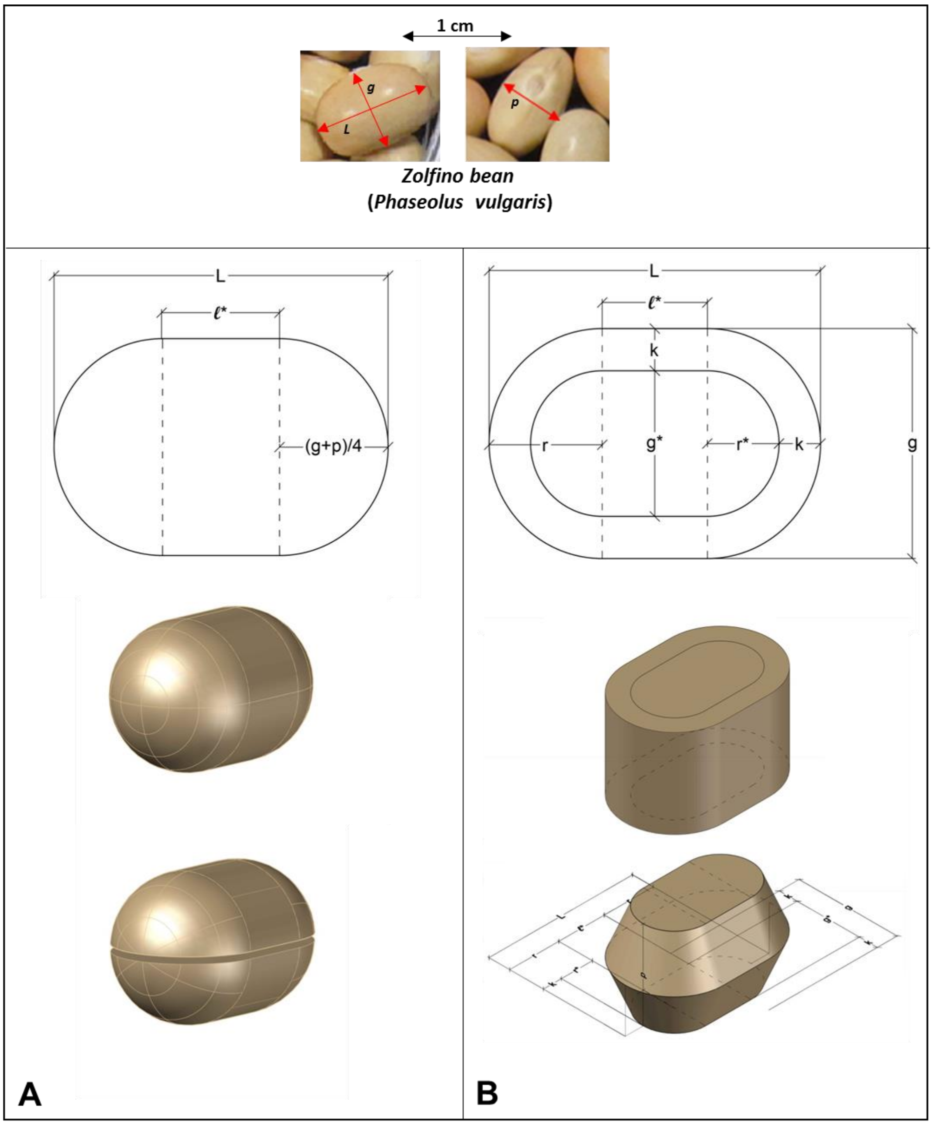

Figure 1S. Graphical representation of the proposed empirical method to evaluate the beans surface areas. Panels A and B refer to Method A and Method B, respectively. The images derived from size parameters measured for Zolfino (See Table 1 and the text for details).

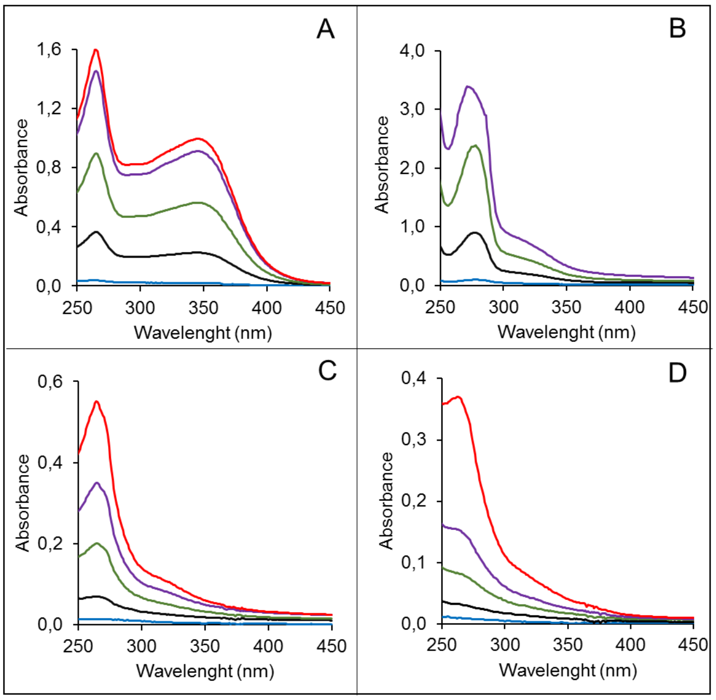

Figure 2S. Extrusion of molecular species from different beans. Zolfino (*Panel A), B*orlotto *(Panel B),* Cannellino *(Panel C)* and Corona *(Panel D)* beans were suspended (0.4 g/mL) in water and the absorption spectra in the UV-visible region of the imbibition media after 0 (blue), 15 (black), 45 (green), 60 (violet) and 120 (red) min of incubation at room temperature were acquired. For all the beans a tenfold dilution in water of the media was performed.

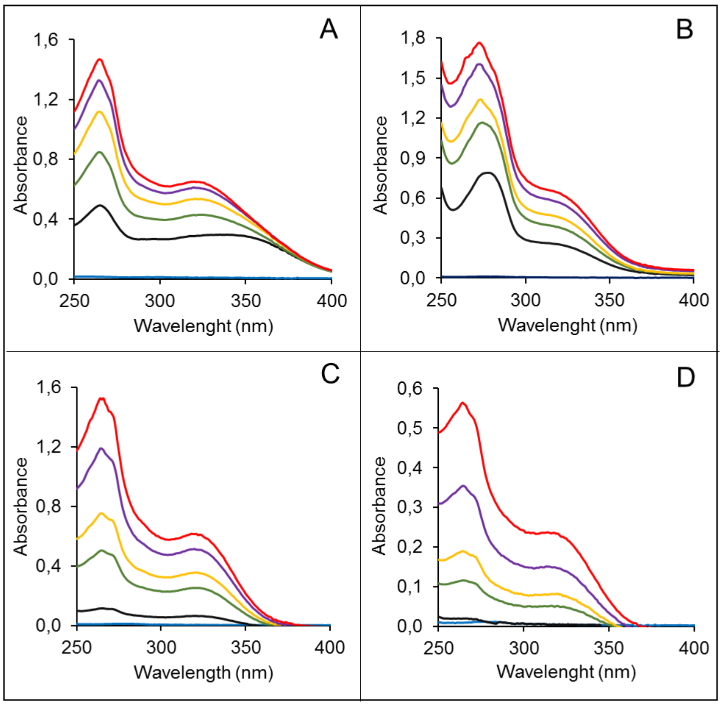

Figure 3S. Extrusion of molecular species from different beans undergoing thermal treatment. Zolfino (*Panel A), B*orlotto *(Panel B),* Cannellino *(Panel C)* and Corona *(Panel D)* beans were suspended (0.4 g/mL) in water and the absorption spectra in the UV-visible region of the imbibition media after 0 (blue), 15 (black), 45 (green), 60 (orange), 90 (violet) and 120 (red) min of incubation at 100 °C were acquired. For all the beans a tenfold dilution in water of the media was performed.

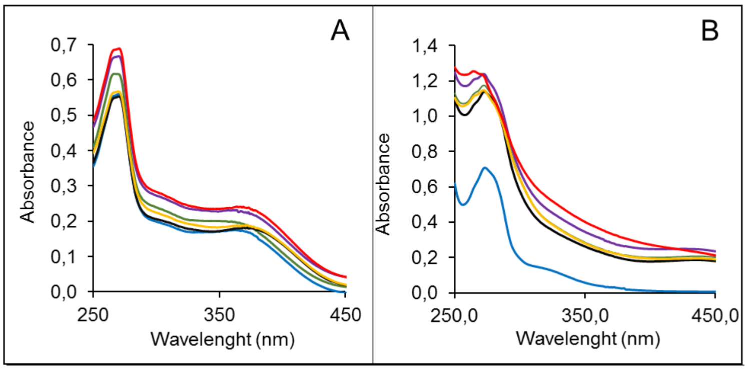

Figure 4S. Thermal treatment of beans imbibition media. The imbibitions media of Zolfino (*Panel A*) and Borlotto (*Panel B*) obtained after an overnight incubation at room temperature were further incubated at 100°C for 0 (blue), 15 (black), 30 (orange), 45 (green), 60 (violet), 90 (red) min and the absorption spectra in the UV-visible region were acquired. For all the beans a tenfold dilution in water of the media was performed.
